# Measuring additive interaction using odds ratios

**DOI:** 10.1186/1742-5573-3-5

**Published:** 2006-04-18

**Authors:** Linda Kalilani, Julius Atashili

**Affiliations:** 1 Department of Epidemiology, School of Public Health, University of North Carolina at Chapel Hill, USA

## Abstract

Interaction measured on the additive scale has been argued to be better correlated with biologic interaction than when measured on the multiplicative scale. Measures of interaction on the additive scale have been developed using risk ratios. However, in studies that use odds ratios as the sole measure of effect, the calculation of these measures of additive interaction is usually performed by directly substituting odds ratios for risk ratios. Yet assessing additive interaction based on replacing risk ratios by odds ratios in formulas that were derived using the former may be erroneous. In this paper, we evaluate the extent to which three measures of additive interaction – the interaction contrast ratio (ICR), the attributable proportion due to interaction (AP), and the synergy index (S), estimated using odds ratios versus using risk ratios differ as the incidence of the outcome of interest increases in the source population and/or as the magnitude of interaction increases. Our analysis shows that the difference between the two depends on the measure of interaction used, the type of interaction present, and the baseline incidence of the outcome. Substituting odds ratios for risk ratios, when calculating measures of additive interaction, may result in misleading conclusions. Of the three measures, AP appears to be the most robust to this direct substitution. Formulas that use stratum specific odds and odds ratios to accurately calculate measures of additive interaction are presented.

## Background

Interaction in epidemiology refers to the extent to which the joint effect of two risk factors on disease differs from the independent effects of each of the factors [1,2]. The joint effect is the effect of the presence of both factors on disease and the independent effect is the effect of each factor in the absence of the other factor. In terms of their causal effects on the incidence of a disease, two risk factors may act independently or interact thereby augmenting (in case of synergism) or reducing (in case of antagonism) the effect of one another. Epidemiologists speak of two distinct types of interaction, biological interaction and statistical interaction. Statistical interaction is a model-dependent concept [3]. It is considered to be present on a multiplicative scale when the joint effect of risk factors differs from the product of the effects of the individual factors [2]. Statistical interaction is present on the additive scale when the joint effect of risk factors differs from the sum of the effects of the individual factors.

Biological interaction on the other hand, describes a property of causality and refers to the interdependent action of two or more factors to produce or prevent an effect [3]. There are limitations in inferring biological/causal interaction based on statistical assessment of interaction. Frequently investigators are not able to identify all of the underlying pathological processes involving measured or unmeasured intervening variables and their effect on the incidence of disease [4]. However, the concept of biological interaction is important in epidemiology for predicting disease on the basis of an individual's profile for a set of risk factors and for planning interventions at the policy level [4]. The appropriate scale on which to statistically assess interaction in such a way that it reflects biological interaction has been a controversial topic in epidemiology. It has been argued that the assessment of interaction on the additive scale is more indicative of the underlying causal mechanism [3,5]. Thus some authors have advocated that the statistical assessment of interaction is best done on the additive scale [3,5-11].

Additive interaction is measured in epidemiological studies primarily using the difference of risk differences also known as the interaction contrast (IC). In cohort studies, this can easily be accomplished by fitting linear or log-linear risk models. However, IC cannot be estimated from case control studies unless the sampling fractions for cases and controls are known or can be estimated [12]. Alternative measures of interaction on the additive scale have been derived from the IC and are based on risks ratios (RR) (see appendix). These measures are, the relative excess risk due to interaction (RERI) also called the interaction contrast ratio (ICR), the attributable proportion due to interaction (AP), and the synergy index (S) [3,6,7,9]. The ICR is the excess risk due to interaction relative to the risk without exposure. AP refers to the attributable proportion of disease which is due to interaction among persons with both exposures. S is the excess risk from exposure (to both exposures) when there is interaction relative, to the excess risk from exposure (to both exposures) without interaction. These measures can be used to assess additive interaction when the odds ratio estimates the risk ratio. However, it is recognized that odds ratios from case-control studies that are not designed to directly estimate the risk or rate ratio, only approximate the risk ratio well when the outcome is rare and do so poorly when the incidence (average risk) of the outcome of interest is high in the source population [6,13-15]. The more frequent the outcome becomes, the more the odds ratio overestimates the risk ratio when it is more than 1 and underestimates the risk ratio when it is less than 1. In addition, the difference between the odds ratio and the risk ratio also depends on the magnitude of the effect of the risk factor. As the effect of a risk factor increases so does the difference between the odds ratio and the risk ratio.

The implications of interpreting measures of interaction on the multiplicative or additive scale when using odds ratios in place of risk ratios have received little attention in the literature. Previous studies have shown that the differences that could arise from assessing interaction using odds ratios instead of risk ratios could be so important as to warrant their careful consideration in epidemiologic research. Morabia *et al *noted that using odds ratios instead of risk ratios can result in discrepant results when assessing statistical interaction on the multiplicative scale [16]. Campbell *et al *further showed that there was a difference in the estimates of interaction assessed both on the additive and multiplicative scale when odds ratios were used in place of risk ratios using ICR [17]. The magnitude of this difference depended both on the baseline risk as well as the magnitude of the joint and independent risk ratios. However, Morabia *et al *only assessed interaction on the multiplicative scale [16] and although Campbell *et al*, assessed interaction on both the additive and multiplicative scale, they only evaluated the performance of ICR and did not include the other measures of interaction, AP and S [17]. In addition, the interaction was assessed within a limited range of effect magnitudes.

All three measures of interaction ICR, AP and S have been used widely in epidemiologic studies [18-26]. However, there have been no studies that have assessed the validity of AP and S as measures of interaction when odds ratios are used in place of risk ratios. In this paper we assess the extent to which the three measures of interaction ICR, AP and S estimated using risk ratios and odds ratios differ as the incidence of the outcome of interest increases in the source population and/or as the magnitude of interaction increases.

## Analysis

### Notation and measures of additive interaction

Throughout this paper the risk of an outcome under the influence of two independent binary exposure variables is considered. The risk of the outcome (the probability of the outcome given an exposure level) is denoted R while the odds of the outcome (the ratio of the probability that the outcome is observed, to the probability of the outcome not being observed given an exposure level) is denoted O. Risk and odds are represented by R_ij _and O_ij _respectively, with i indexing exposure to the first variable, and j indexing exposure to the second variable. The subscripts i and j take values of 0 or 1 in the absence or presence of exposure respectively. Thus R_11 _stands for the risk of the outcome when doubly exposed, while O_10 _stands for the odds of the outcome given exposure to the first factor and non-exposure to the second factor. Similarly, using RR and OR to denote risk ratio and odds ratio respectively and using doubly unexposed to both factors as the reference, RR_01 _stands for the RR comparing the risk of the outcome in subjects exposed to the second factor but not to the first, to the risk in the doubly unexposed (R_01_/R_00_) while OR_11 _stands for the OR comparing the doubly exposed to the doubly unexposed (O_11_/O_00_).

Additive interaction is assessed using the three measures proposed by Rothman [3,9] – the interaction contrast ratio (ICR), the attributable proportion due to interaction (AP) and Rothman's synergy index (S). In this paper, when these are calculated using the RR as suggested by Rothman, they are referred to as ICR, AP and S. However, when they are calculated by substituting ORs for RRs they are referred to as ICRF, APF and SF respectively (F stands for 'false' as these are false measures of ICR, AP and S). Formulas for these measures are given in the appendix.

### Simulations and assumptions

Using SAS statistical software version 9 (by the SAS Institute Inc. Cary, NC, USA) risks for the doubly exposed (R_11_) and the doubly unexposed (R_00_) portions of 100,000 scenarios were simulated using a random uniform distribution of risks ranging from 0 to 1. The simulations allowed us, not only to examine a wide range of combinations of the baseline risk and magnitude of interaction, but they also permitted us to describe the shape and magnitude of discrepancy between the interaction measures using RR and OR. To simplify the comparison of the measures of interaction, the effects of each of the exposures were fixed to 2 such that in each cohort RR_10 _= RR_01_= 2. Thus to avoid implausible values of R_01 _and R_10 _(risk values greater than 1) this analysis was limited to simulated cohorts in which the baseline risks (R_00_) were less than 0.5. This analysis was also restricted to that of two causative factors such that only cohorts where R_01 _and R_01 _were both greater than R_00 _were included. This did not preclude R_11 _from being less than R_00 _and/or R_10 _(or R_01_) thus allowing for cohorts in which the risk factors were antagonistic to each other. We also assumed complete assessment of the population such that risks and odds (and consequently risk ratios and odds ratios) were assessed without sampling error. Finally, the absence of any confounders of the effect of each of the factors in all the cohorts was also assumed.

## Results

**Figure 1:**
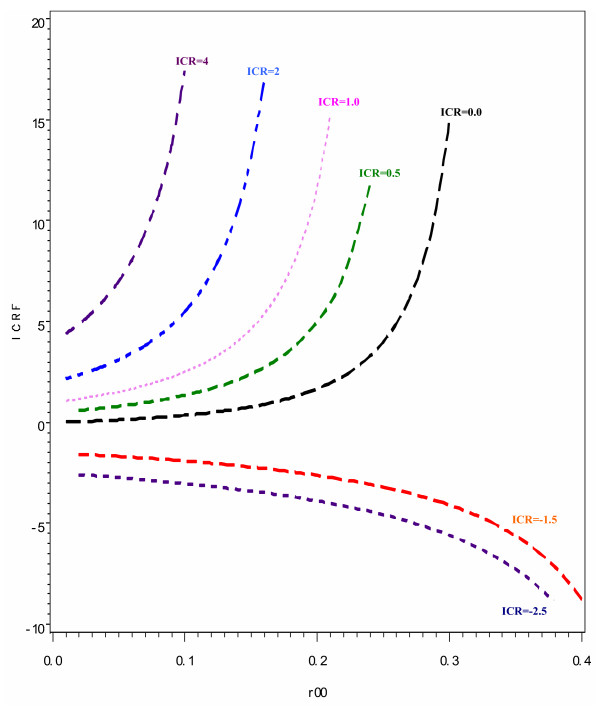
**The impact of baseline risk (R_00 _and interaction magnitude on the discrepancy between the interaction contrast ratio calculated using risk ratios (ICR) and that calculated using odds ratios (ICRF). Each curve represents a specific value of the ICR and changes on the vertical scale represent the magnitude of the discrepancy**. Interaction scenarios: Less than additive; ICR = -2.5 and -1.5; Exactly additive: ICR = 0; More than additive and less than multiplicative ICR = 0.5; Exactly multiplicative: ICR = 1; More than additive and more than multiplicative: ICR = 2 and 4

Figure 1 depicts the values of ICRF which represent the estimates of the ICR when odds ratios were used instead of risk ratios at different baseline risks. If using odds ratios to assess interaction had resulted in the same estimates as would have been obtained using risk ratios, the values of ICRF would have been the same as the ICR values. Hence we would have expected to get horizontal lines in all five scenarios of interaction and at all levels of the baseline risk. However, as illustrated in Figure 1, as the baseline risk increases, the ICRF values diverge from the expected values of ICR and this discrepancy is more evident as the baseline risk approaches 0.15. When there is no interaction between the two risk factors, i.e. when their joint effect is the exact additive of the two individual effects, the ICR has a value of 0. However, when the risk ratios are replaced by the odds ratios, the ICRF has values that appreciably overestimate ICR thus indicating the presence of interaction. This is noticeable starting from when the baseline risk approaches 0.10, becoming very prominent at a baseline risk level of 0.30 at which point the value of ICRF is 15. In these instances, one would conclude that there is evidence of interaction on the additive scale even though the same data would not suggest the presence of additive interaction had risk ratios been used. This discrepancy in the estimates of ICR and ICRF is also more pronounced when the interaction is more than additive as compared to when the interaction is less than additive. When the interaction is more than additive and more than multiplicative, the difference in the values of ICR and ICRF becomes evident even at baseline risks as low as 0.05. At this baseline risk, the value of ICRF is 7, which is almost double the estimate that would have been obtained if risk ratios had been used instead to give an ICR value of 4. However, when the interaction is less than additive, the difference in the ICR and the ICRF estimates is not very evident until the baseline risk is approximately 0.20. In the scenario where the ICR value is -1.5, the ICRF estimate is -1.9, which is not very different from the ICR value at a baseline risk of 0.10. However, this discrepancy increases markedly such that at a baseline risk of 0.20, the ICRF has a value -2.6. Figure 4 also illustrates the magnitude of the differences between ICR and ICRF for different combinations of baseline risk and interaction magnitude.

**Figure 2:**
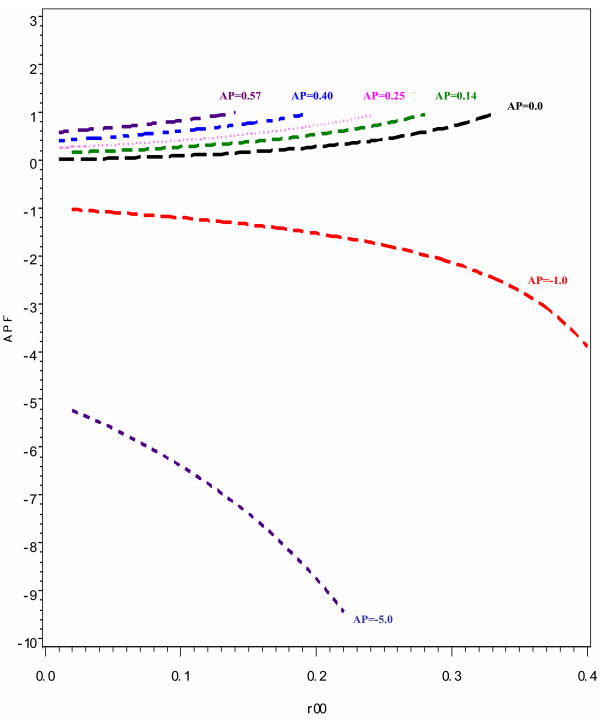
**The impact of baseline risk (R_00 _and interaction magnitude on the discrepancy between the attributable proportion due to interaction calculated using risk ratios (AP) and that calculated using odds ratios (APF). Each curve represents a specific value of AP and changes on the vertical scale represent the magnitude of the discrepancy**. Interaction scenarios: Less than additive; **AP **= 5 and -1; Exactly additive: **AP **= 0; More than additive and less than multiplicative **AP **= 0.14; Exactly multiplicative: **AP **= 0.25; More than additive and more than multiplicative: **AP **= 0.40 and 0.57 (These correspond to the ICR values depicted in Figure 1).

A similar relationship is noted when additive interaction is assessed using AP. As illustrated in the Figure 2, the difference in the magnitude of APF from AP increases with an increase in the baseline risk. However, this difference only becomes appreciably evident at a baseline risk of approximately 0.15, which is higher than that seen when using ICR. At the baseline risk of 0.05, there is a small difference in the AP and APF values. The AP values of 0, 0.25 and 0.40 have corresponding APF values of 0.03, 0.32 and 0.49 respectively. However, at the baseline risk of 0.15, the differences in magnitude are noticeable as the corresponding APF values are 0.06, 0.55 and 0.77 respectively. Furthermore, the differences in the estimates of AP and APF are not as pronounced when the interaction is more than additive, as was the case when using ICR to measure interaction. However, as opposed to using ICR, the difference is more pronounced when the interaction is less than additive. When the value of AP is -1, the difference in the estimates of AP and APF are not substantial even at baseline risk levels of 0.25 where the value of APF is -1.8. However, when the magnitude of the less than additive interaction between the two risk factors increases and the AP value is -5, the difference in the estimates of AP and APF become very marked even at a lower baseline risk of approximately 0.05 such that the value of APF is 6.4.

**Figure 3:**
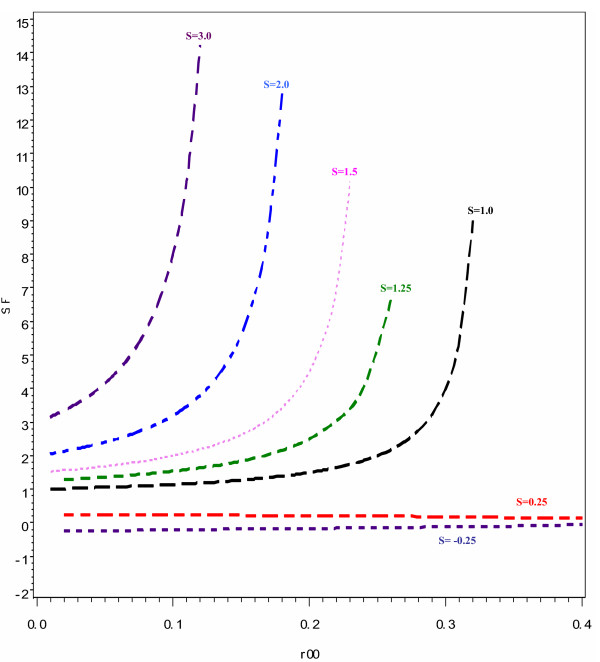
**The impact of baseline risk (R_00 _and interaction magnitude on the discrepancy between the synergy index calculated using risk ratios (S) and that calculated using odds ratios (SF). Each curve represents a specific value of S and changes on the vertical scale represent the magnitude of the discrepancy**. Interaction scenarios: Less than additive; **S **= -0.25 and 0.25; Exactly additive: **S **= 1; More than additive and less than multiplicative **S **= 1.25.; Exactly multiplicative: **S **= 1.5; More than additive and more than multiplicative: **S **= 2 and 3 (These correspond to the ICR values depicted in Figure 1).

As illustrated in Figure 3, a similar pattern is also observed when assessing the presence of additive interaction using S. The discrepancy in the values of S and SF increases when the baseline risk increases and the magnitude of interaction increases. When there is exact additivity, indicating no interaction on the additive scale, S has a value of 1. However, there is divergence of the values of SF from S, in the scenario where we would have expected exact additivity if risk ratios had been used. This is appreciably evident when the baseline risk is approximately 0.25 where the value of SF is 1.5 instead of 1. This noticeable difference is at a much higher baseline risk than that seen when the two previous measures of interaction, ICR and AP were used. As was noted in the case of ICR, the differences in the estimates of S and SF become more evident at lower levels of baseline risk when the is interaction is greater than additive. For example, when the interaction is more than additive and more than multiplicative, the difference in the two estimates is appreciable even at baseline risks as low as 0.05 such that the value of SF is 4.2, instead of an S value 3. Because of the predetermined risk ratio of 2 for both risk factors, for our simulations the lowest value that S can have is -0.5. When there is less than additive interaction the discrepancy in the values of S and SF decreases as the value of S approaches 1.

**Figure 4:**
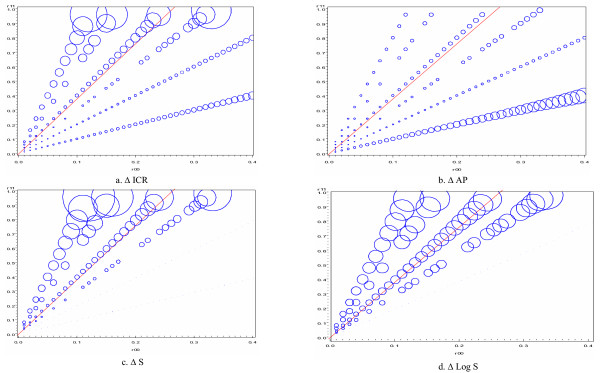
**Discrepancy between ICR, AP, S and logS calculatedusing the RR and the OR for different values of R_11 _and R_00_**. The size of each bubble is directly proportional to the absolute difference in the interaction measures calculated using odds ratios and risk ratios at each value of R_11 _and R_00_. The line in each plot represents the set of R_11 _and R_00 _scenarios in which there is exact additivity (ICR = AP = 0; S = 1).

The effect of calculating the three interaction measures using odds ratios instead of risk ratios is further illustrated in Figure 4. The size of the bubbles represents the magnitude of the difference between the interaction measures using odds ratios and risk ratios. The lines represent the scenario when there is exact additivity. Panel 4a (left upper corner) represents the absolute difference in the values of ICR and ICRF. It can be noted that the difference in these estimates increases with an increase in the baseline risk. In addition, the difference is more evident in the presence of interaction that is more than additive compared to the other types of interaction as indicated by the size of the bubbles.

Panel 4b (right upper corner) is a representation of the estimates of interaction calculated using AP and APF. As was the case for ICR/ICRF, the differences in the absolute values increase as the baseline risk increases. However, in this instance the increase in the discrepancy between AP and APF is more gradual in contrast to ICR/ICRF, and the difference is more prominent when the interaction is less than additive. Panel 4c (bottom left corner) represents the absolute difference between S and SF and Panel 4d (bottom right corner) represents the log difference between S and SF. The pattern is very similar to that seen when the presence of interaction was assessed using ICR/ICRF. The difference increases as the baseline risk increases and it is more prominent when there is interaction that is more than additive.

## Discussion

Our analysis shows that measures of additive interaction calculated by substituting odds ratios in place of risk ratios may not be reliable. The discrepancy between the two depends on the measure of interaction used, the type of interaction present and the baseline risk of the outcome. For more than additive interactions the difference is more pronounced for the ICR and S and the least difference is noticed for AP. For less than additive interactions, the least difference is noticed for ICR and S but it is more pronounced for AP. For all of the three measure of interaction, when odds ratios were substituted in the place of risk ratios, the difference in magnitude increased with an increase in the baseline risk and magnitude of interaction. Additionally, for all three measures, interaction assessed to be more (or less) than additive using the RR was always found to be also more (or less) than additive, albeit of higher absolute magnitude, when the OR was used. However, an absence of additive interaction (exact additivity) could be misleadingly assessed as the presence of an interaction when using the OR. There was no cross over, that is, never did a more than additive interaction appear to be less than additive and vice versa. Thus when evaluating the interaction between two risk factors using the OR and using the ICR or S as measure of interaction, investigators should be cautious about affirming the presence of more than additive interaction, particularly when the study outcome is not rare. Similarly, investigators should be careful when interpreting less than additive interaction based on OR, particularly if AP is used.

Morabia *et al*. previously described a similar discrepancy between the OR and RR when assessing interaction but this was limited to interaction on the multiplicative scale [16]. Campbell *et al *also reported similar discrepancies both on the multiplicative scale and on the additive scale using the ICR [17]. Although most authors tend to assess interaction on the multiplicative scale when using ORs, particularly in the context of case-control study designs and/or logistic regression analysis [22-24,26], in this paper we only evaluated the assessment of interaction on the additive scale. In simulations using individual level etiologic models (such as the Potential Outcomes Model and the Sufficient and Component Cause Model), the additive scale appeared to be a better indicator of the relative frequency of antagonistic or synergistic interactions in the study population [3. 5]. Thus the additive scale has been argued to be the scale of choice to assess interaction.

The three measures ICR, AP and S were developed independently. ICR and AP were derived directly from IC which is the primary measure of additive interaction as illustrated in the formulae in the appendix. ICR expresses IC as a proportion to the baseline risk (R_00_) and AP expresses IC as a proportion of the joint effect of the two risk factors (R_11_). S is the ratio of the observed and expected effects of the difference between the joint effect and the baseline risk. Of the three measures of interaction on the additive scale (ICR, AP, and S), the ICR appears to be most frequently utilized [3,25,27]. However, ICR may not be the best measure to use for assessing interaction in all circumstances. Our study suggests that AP is more robust to directly substituting ORs in place of RRs. It is less affected, compared to ICR and S, by changes in the type of interaction or baseline risk when substituting risk ratios by odds ratios. Furthermore, in the analysis of additive interaction while adjusting for confounding (using additional covariates in regression models), S has been shown to be the most reliable as it does not vary across strata of the additional covariates [12]. We suggest that all three measures be used when assessing additive interaction and that any discrepancies between the three should be resolved by a careful consideration of the individual risk factors being evaluated, the baseline risk of the outcome, as well as the presence or not of confounders.

Extensive work has been done to accommodate odds ratios in epidemiologic studies [28]. However, to the best of our knowledge, these do not address the measurement of additive interaction when left only with odds ratios as measures of effect. Campbell *et al *provide a formula that shows how a measure of multiplicative interaction based on risk ratios can be estimated using odds ratios and baseline risk [17]. We have derived formulas to calculate the three additive interaction measures using odds ratios. The formulas presented in the appendix could be used to more accurately calculate the three additive measures of interaction, similar to what would have been obtained, had the risk ratio been used. Odds for each level defined by combinations of exposure and covariate can be estimated using parameters from a logistic regression. The formulas could easily be implemented using a spreadsheet (see Additional File 1).

## Conclusion

Substituting ORs for RRs, when calculating measures of additive interaction (ICR, AP and S), may result in misleading conclusions. The magnitude of the discrepancy in the measures of interaction increases with an increase in the baseline incidence of the outcome and depends on the measure of interaction used as well as the type of interaction. We recommend that formulas modified for direct use of odds and odds ratios be employed for more accurate assessment of additive interaction.

## Appendix

### I Measures of additive interaction

The interaction contrast (IC) which is the basic measure of additive interaction is applicable when the risks for each stratum defined by 2 variables are available. It is given by:

IC = R_11 _- R_10 _- R_01 _+ R_00 _   Eq. 1

However when only the risk ratios (and not the risks for each level defined by exposure-covariate combinations) are available, the following extensions of IC can be used to evaluate additive interaction [3,9]

IC = R_11 _- R_10 _- R_01 _+ R_00_







ICR and AP thus have a null value of 0 while S has a null value of 1. With some study designs, such as cross-sectional studies or case-control studies, the only measure of effect that can be obtained is the odds ratio, OR. To evaluate interaction on the additive scale some authors have simply substituted OR for RR in equations 2–4. We refer to ICR, AP and S calculated using the OR as ICRF, APF, and SF respectively. If the OR is not a good estimate of the RR then ICRF, APF and SF may not be good estimates of ICR, AP and S respectively.

To correctly calculate ICR, AP and S, from studies in which only the OR is available one simply needs to calculate the RR based on the OR and substitute it in equations 2–4 (instead of directly substituting OR for RR).

To calculate the RR using the OR, consider a single binary exposure scenario:





Thus



Extending equation 7 to the two binary exposure variable scenario then







Substituting equations 8–10 in equations 2–4:











Thus, to more accurately calculate ICR, AP and S, the odds of the outcome for each level defined by combinations of the 2 covariates being evaluated for interaction, need to be known.

These odds can be estimated using parameters from a logistic regression and sampling probabilities (when needed) as follows {[3] pp 416–422}:



Where

k is the number of exposure levels defined by combinations of covariates (for example for 2 binary covariates, k = 4);

W_j _are mutually exclusive indicator variables for each of the k-l exposure levels (excluding a referent level);

β_j _are the estimated log odds ratios comparing each index j level to the referent level;

α* is the estimated intercept parameter from the logistic regression;

f and g are case and control sampling probabilities respectively.

Note that equation 16 is based on the fact that in case-control studies, the intercept from a logistic regression model is the log of the odds of the outcome inflated by the log of the ratio of case to control sampling probabilities [12].

In cohort studies, cross sectional studies, or case control studies with complete ascertainment of both cases and controls, in which analysts use logistic regression, f = g = 1, and equation 16 reduces to equation 17.



Equations 12, 14 and 15 can be easily implemented using a spreadsheet (see Additional File 1).

### II. SAS code

data one simulate;

   do sample = 1 to 100000;

   r00 = round((0.00001 + ranuni(11)),.01);

   r11 = round ((0.00001 + ranuni(14)), 0.01);

   r01 = .;

   if r00<0.5 then r01 = 2*r00;

   r10 = .;

   if r00<0.5 then r10 = 2*r00;

   rr11 = r11/r00;

   rr10 = r10/r00;

   rr01 = r01/r00;

   or11 = (r11*(1-r00))/(r00*(1-r11));

   or01 = (r01*(1-r00))/(r00*(1-r01));

   or10 = (r10*(1-r00))/(r00*(1-r10));

   icr = round(((r11/r00)-(r10/r00)-(r01/r00)+1), .01);

   ap = round(((r11/r00)-(r01/r00)-(r10/r00)+1)/(r11/r00), .01);

   s = round(((rr11-1)/(rr10 + rr01 -2)), .01);

   icrf = round((or11- or10- or01 + 1), .01);

   apf = round((or11-or01-or10+1)/or11, .01);

   sf = round((or11-1)/(or10 + or01 -2), .01);

   icrdif = abs(icrf-icr);

   apdif = abs(apf-ap);

   sdif = abs(sf-s);

   logsdif = abs(log(sf) – log(s));

   output simulate;

   end;

   **run;**

**proc sort **data = simulate; by r00 ; **run;**

axis1 order = -10 to 20 by 5 label = (a = 90 r = 0 'ICRF' h = 2);

axis2 order = 0 to 0.4 by 0.1;

**procgplot **data = simulate ; where icr in (-2.5,-1.5, 0,0.5,1, 2, 4,);

symbol1 i = spline v = star h = 0.5 c = black;

symbol2 i = spline v = plus h = 0.5 c = red;

symbol3 i = spline v = circle h = 0.5 c = blue;

symbol4 i = spline v = diamond h = 0.5 c = green;

symbol5 i = spline v = square h = 0.5 c = violet;

symbol6 i = spline v = triangle h = 0.5 c = indigo;

symbol7 i = spline v = dot h = 0.5 c = black;

plot icrf*r00 = icr/haxis = axis2 vaxis = axis1 nolegend;

run;quit;

axis1 order = -10 to 3 by 1 label = (a = 90 r = 0 'APF' h = 2);

axis2 order = 0 to 0.4 by 0.1;

**proc gplot **data = simulate ;

where icr in (-2.5,-1.5, 0, 0.5,1, 2, 4,);

symbol1 i = spline v = star h = 0.5 c = black;

symbol2 i = spline v = plus h = 0.5 c = red;

symbol3 i = spline v = circle h = 0.5 c = blue;

symbol4 i = spline v = diamond h = 0.5 c = green;

symbol5 i = spline v = square h = 0.5 c = violet;

symbol6 i = spline v = triangle h = 0.5 c = indigo;

symbol7 i = spline v = dot h = 0.5 c = black;

plot apf*r00 = ap/haxis = axis2 vaxis = axis1 nolegend;

run;quit;

axis1 order = -5 to 15 by 1 label = (a = 90 r = 0 h = 1 'SF' h = 2);

axis2 order = 0 to 0.4 by 0.1;

**proc gplot **data = simulate ;

where icr in (-2.5,-1.5, 0, 0.5,1, 2, 4,);

symbol1 i = spline v = star h = 0.5 c = black;

symbol2 i = spline v = plus h = 0.5 c = red;

symbol3 i = spline v = circle h = 0.5 c = blue;

symbol4 i = spline v = diamond h = 0.5 c = green;

symbol5 i = spline v = square h = 0.5 c = violet;

symbol6 i = spline v = triangle h = 0.5 c = indigo;

symbol7 i = spline v = dot h = 0.5 c = black;

plot sf*r00 = s/haxis = axis2 vaxis = axis1 nolegend;

run;quit;

*bubble plots;

**data **anno2;/*this superimposes a line where there is exact additivity ie icr = 0, rr11 = 3 */function = 'move'; xsys = '1'; ysys = '1'; x = 0; y = 0; output;

function = 'draw'; xsys = '1'; ysys = '1'; color = 'red'; x = (100/3)*2; y = 100; output;

run;

axis1 order = 0 to 1 by .1;

axis2 order = 0 to 0.4 by 0.1;

**proc gplot **data = simulate ;

where icr in (-3, -2, -1, 0, 1,3, 5);

bubble r11*r00 = icrdif/anno = anno2 haxis = axis2 vaxis = axis1 bsize = 100 bcolor = blue;

bubble r11*r00 = apdif/anno = anno2 haxis = axis2 vaxis = axis1 bsize = 100 bcolor = blue;

bubble r11*r00 = sdif/anno = anno2 haxis = axis2 vaxis = axis1 bsize = 100 bcolor = blue;

bubble r11*r00 = logsdif/anno = anno2 haxis = axis2 vaxis = axis1 bsize = 100 bcolor = blue;

run;quit;

## Abbreviations

R: Risk

O: Odds

RR: Risk ratio

OR: Odds ratio

RERI: Relative excess risk due to interaction

ICR: Interaction contrast ratio

AP: Attributable proportion due to Interaction

S: Rothman's synergy index

ICRF: Interaction contrast ratio calculated by substituting odds ratios for risk ratios

APF: Attributable proportion due to interaction calculated by substituting odds ratios for risk ratios

SF: Rothman's synergy index calculated by substituting odds ratios for risk ratios

## Competing interests

The author(s) declare that they have no competing interests.

## Authors' contributions

LK conceived the research question. LK and JA designed and conducted the analysis. Both authors wrote the manuscript.

## Supplementary Material

Additional File 1 Spreadsheet for calculating measures of additive interaction (ICR, AP and S) using odds. Click here for file
